# Flucloxacillin and cefazolin for treatment of *Staphylococcus aureus* bloodstream infection

**DOI:** 10.1007/s15010-023-02168-8

**Published:** 2024-01-31

**Authors:** Kirsten Schmidt-Hellerau, Marianne Breuninger, Johanna Kessel, Maria J. G. T. Vehreschild, Gregor Paul, Jomana Reusch, Norma Jung, Martin Hellmich, Gerd Fätkenheuer

**Affiliations:** 1 Department I of Internal Medicine, Infectious Diseases, Medical Faculty, University Hospital of Cologne, University of Cologne, Cologne, Germany; 2 Department of Internal Medicine, Infectious Diseases, Goethe University Frankfurt, University Hospital Frankfurt, Frankfurt am Main, Germany; 3 Institute of Medical Statistics and Computational Biology (IMSB), Faculty of Medicine, University Hospital Cologne, University of Cologne, Cologne, Germany

**Keywords:** *Staphylococcus aureus*, MSSA, Bacteraemia, Cefazolin, Flucloxacillin, Antistaphylococcal penicillins

## Abstract

**Purpose:**

Antistaphylococcal penicillins and cefazolin have been used as first line therapy in Methicillin-susceptible Staphylococcus aureus bloodstream infection. While efficacy of both regimens seems to be similar, the compounds may differ with regard to tolerability. This study aims to describe the clinical use of cefazolin and flucloxacillin, focussing on discontinuation or change of anti-infective agent due to adverse events.

**Methods:**

This observational prospective study was conducted at two German tertiary care centres with an internal recommendation of flucloxacillin for MSSA-BSI in one, and of cefazolin in the other centre. Adverse events were registered weekly under treatment and at a 90-day follow-up. Descriptive analysis was complemented by a propensity score analysis comparing adverse events (stratified rank-based test applied to the sum of Common Terminology Criteria for adverse events ratings per patient).

**Results:**

Of 71 patients included, therapy was initiated with flucloxacillin in 56 (79%), and with cefazolin in 15 (21%). The propensity score analysis indicates a statistically significant difference concerning the severity of adverse events between the treatment groups in favour of cefazolin (*p* = 0.019). Adverse events led to discontinuation of flucloxacillin in 7 individuals (13% of all patients receiving flucloxacillin). Clinical outcome was not different among treatment groups.

**Conclusion:**

Using cefazolin rather than flucloxacillin as a first line agent for treatment of MSSA-BSI is supported by these clinical data.

## Background

Methicillin-susceptible *Staphylococcus aureus* (MSSA) blood stream infection (BSI) is traditionally treated with antistaphylococcal penicillins (ASP). Alternative treatment with cefazolin has been recommended, for example, as first choice in penicillin allergy in infectious endocarditis [1, 2], as well as in catheter related bloodstream infections in dialysis patients [3]. During recent years, cefazolin has been increasingly investigated as first line treatment of MSSA BSI. Theoretical concerns regarding the Cefazolin inoculum effect, an in-vitro phenomenon of reduced susceptibility at high bacterial density that has been described in 13–58% of *S. aureus* isolates, have not been shown to be of overall clinical relevance, except possibly for subgroups of patients infected with certain *S. aureus* strains [4, 5]. Regarding the question if Cefazolin should be used for central nervous system infections, a recent review concludes Cefazolin being a reasonable option for a variety of CNS infections [6]. Cefazolin has been attributed less side effects than ASP and seems to result in health care cost savings [7]. Reviews and meta-analyses have suggested that cefazolin is at least as effective as ASP while possibly being safer and associated with less acute kidney injury [8–11]. However, due to the partly low quality of trials and their mostly retrospective nature, this has not yet led to substantial change in treatment guidelines. This prospective study aims to gain an insight into the spectrum of AE of cefazolin and flucloxacillin, as well as into clinical decisions regarding treatment changes due to AE.

## Methods

This prospective observational study was conducted at two university hospitals in Germany between June 2019 and May 2021, with an internal recommendation of flucloxacillin for MSSA-BSI in one, and of cefazolin in the other centre. At both sites, dose recommendations were Flucloxacillin 12 g daily (continuously or divided in 3–4 doses) or Cefazolin 2 g IV three times daily, adjusted to renal function if necessary. Treatment duration was defined by the treating physician, based on type of infection and current medical standards. Inpatients with confirmed monobacterial MSSA BSI and cefazolin or flucloxacillin as part of current or planned antibiotic treatment were included, if they were able to provide written informed consent. Both sites have an active infectious disease consulting service, which is regularly involved in treatment of patients with *S. aureus* infections and identified patients suitable for this study. Data was collected on age, gender, pre-existing comorbidities and presumed focus of infection. During the course of antibiotic treatment, weekly clinical follow-up visits were conducted to register adverse events (AE) and changes in the therapeutic regimen. After 90 days, patients were contacted by phone for follow-up data on recurrent infection and mortality.

Data were analyzed using SPSS Statistics (IBM Corp., Armonk, NY, USA). Descriptive statistics of the cohort (age, sex, comorbidities) and antibiotic treatment of MSSA included absolute and relative frequencies. The main outcome was discontinuation or change of antibiotic treatment with cefazolin or flucloxacillin due to AE. Further, number and severity of AE, end-of-treatment clinical and microbiological response, as well as 90-day MSSA associated mortality and recurrent infection were described. Finally, the number and severity of side effects of flucloxacillin and cefazolin were compared using a propensity score approach. Here, the propensity score (PS) is the probability of a person receiving cefazolin. In the corresponding logistic regression analysis, the covariates of gender, age, and Charlson Comorbidity Index were utilized as predictors. We created four PS strata (quartiles) where the two treatment groups share similar characteristics (“quasi-randomised”) and stratified a rank-based analysis (i.e., log-rank test) accordingly. Observations from patients receiving both drugs successively were treated as approximately independent. The number of these patients (*n* = 7) is too small to successfully adjust for nesting or correlation.

## Results

Of 71 patients included, 56 (79%) received flucloxacillin as initial therapy of *S. aureus* BSI, and 15 (21%) cefazolin. Two thirds of patients were between 50 and 79 years old (median 61 years; interquartile range 53–72 years). The Charlson Comorbidity Index indicated moderate or severe comorbidity in 33% (*n* = 5/15) of patients started on cefazolin, and 49% (*n* = 30/56) of patients started on flucloxacillin. See Table 1 for a description of the cohort. In a quarter of patients, MSSA BSI was hospital acquired (*n* = 17/67, 25%). See Table 2 for the sites of infection.

**Table 1:** Patient characteristics of total cohort and per substance used for initial targeted therapy

	Total cohort (*n* = 71)	Flucloxacillin as initial targeted therapy (*n* = 56)	Cefazolin as initial targeted therapy (*n* = 15)
	*N* (%)	*N* (%)	*N* (%)
Age (years)			
21–40	6 (9)	3 (5)	3 (20)
41–60	28 (39)	23 (41)	5 (33)
61–80	31 (44)	24 (43)	7 (47)
> 80	6 (9)	6 (11)	0 (0)
Gender			
Male	49 (69)	38 (68)	11 (73)
Female	22 (31)	18 (32)	4 (27)
Comorbidity			
Myocardial infarction	11 (16)	9 (16)	2 (13)
Chronic heart failure	17 (24)	12 (21)	5 (33)
Peripheral artery disease	8 (11)	5 (9)	3 (20)
Cerebrovascular disease	14 (20)	13 (23)	1 (7)
Dementia	1 (1)	1 (2)	0 (0)
Rheumatologic disease	5 (7)	5 (9)	0 (0)
Peptic ulcer disease	5 (7)	3 (5)	2 (13)
Moderate/severe liver disease	4 (6)	4 (7)	0 (0)
Chronic pulmonary disease	10 (14)	9 (16)	1 (7)
Moderate/severe renal disease	12 (17)	9 (16)	3 (20)
Diabetes mellitus with end organ damage	9 (13)	7 (13)	2 (13)
Haematological malignancy	7 (10)	7 (13)	0 (0)
Solid tumour (within the last 5 years)	8 (11)	6 (11)	2 (13)
Metastatic tumour	9 (13)	8 (14)	1 (7)

**Table 2:** Site(s) of infection

	Total cohort (*n* = 70)^1^	Flucloxacillin (*n* = 49)^1^	Cefazolin (*n* = 14)	Flucloxacillin followed by cefazolin (*n* = 6)	Cefazolin followed by flucloxacillin (*n* = 1)
	*N* (%)	*N* (%)	*N* (%)	*N* (%)	*N* (%)
Peripheral line	21 (30)	14 (29)	3 (21)	4 (67)	0 (0)
Soft tissue infection	16 (23)	12 (24)	4 (29)	0 (0)	0 (0)
Central line	9 (13)	7 (14)	2 (14)	0 (0)	0 (0)
Infectious endocarditis	8 (11)	5 (10)	3 (21)	0 (0)	0 (0)
Spondylodiscitis	7 (10)	5 (10)	1 (7)	0 (0)	1 (100)
Periprosthetic joint infection	4 (6)	3 (6)	1 (7)	0 (0)	0 (0)
Septic arthritis	3 (4)	2 (4)	0 (0)	1 (17)	0 (0)
Other^2^	7 (10)	6 (12)	1 (7)	0 (0)	0 (0)
Unknown	3 (4)	2 (4)	1 (7)	1 (17)	0 (0)

^1^*n* = 1 missing

^2^Pleural empyema, pulmonary septic emboli, dental abscess, neurostimulation device, left ventricular assist device, surgical site infection, osteomyelitis

In patients receiving flucloxacillin, 182 AE were reported (3.2 per patient), and 30 in patients receiving cefazolin (1.4 per patient). The types of AE are presented in Table 3. Of these AE, 16 were severe (Common Terminology Criteria for Adverse Events (CTCAE) grade 3 or 4) in patients receiving flucloxacillin (*n* = 16/57, 28%), and 6 in patients receiving cefazolin (*n* = 6/21, 29%). AE classified as severe (grade 4) were leukopenia and hypopotassaemia in one patient each while receiving flucloxacillin, and dizziness, abdominal pain and phlebitis in one patient receiving cefazolin.

**Table 3:** Reported AE while receiving flucloxacillin or cefazolin

	Flucloxacillin (*n* = 57)	Cefazolin (*n* = 21)	Total (*n* = 78)
	*N* (%)	*N* (%)	*N* (%)
Abdominal pain	8 (14)	3 (14)	11 (14)
Allergic reaction	9 (16)	1 (5)	10 (13)
Anaemia	5 (9)	0 (0)	5 (6)
Diarrhoea	10 (18)	4 (19)	14 (18)
Elevated liver enzymes	10 (18)	1 (5)	11 (14)
Elevated renal function parameters	12 (21)	2 (10)	14 (18)
Fever	8 (14)	1 (5)	9 (12)
Headache	12 (21)	2 (10)	14 (18)
Hypopotassaemia	14 (25)	0 (0)	14 (18)
Leukopenia	4 (7)	0 (0)	4 (5)
Nausea	20 (36)	3 (14)	23 (29)
Peripheral oedema	27 (48)	3 (14)	30 (38)
Phlebitis	22 (39)	4 (19)	26 (33)
Sweats	1 (2)	0 (0)	1 (1)
Vaginal yeast infection	3 (5)	0 (0)	3 (4)
Vertigo/dizziness	6 (11)	2 (10)	8 (10)
Visual impairment	1 (2)	0 (0)	1 (1)
Vomiting	10 (18)	4 (19)	14 (18)

**Fig. 1:**
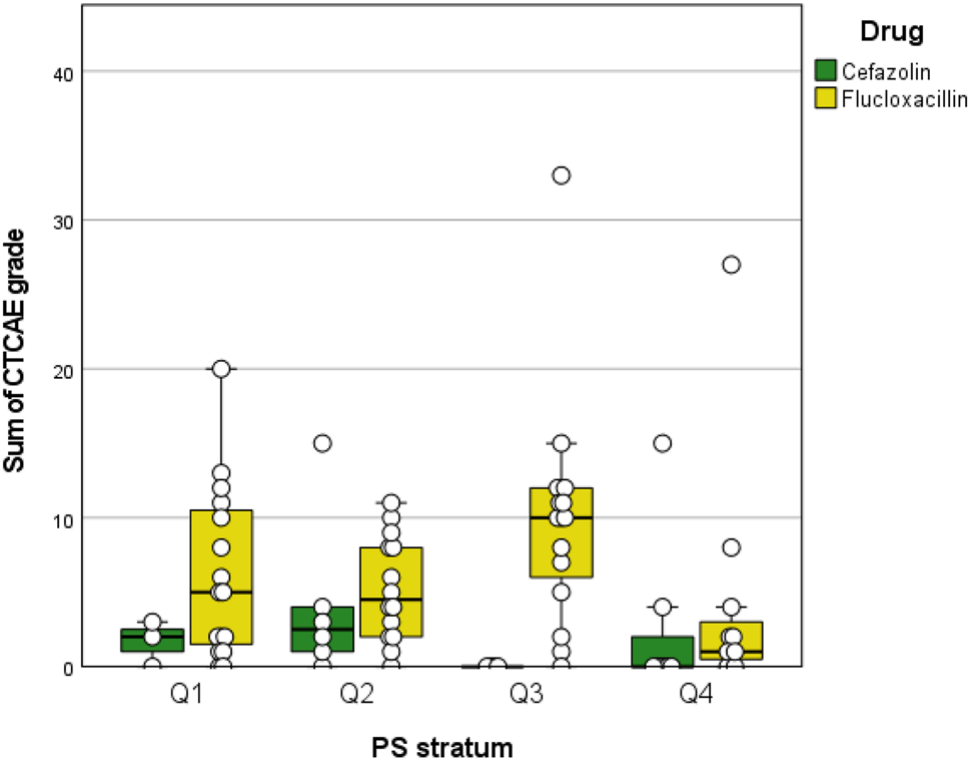
Boxplots of stratified propensity score groups, illustrating the differences in the sum of CTCAE ratings per patient across treatment cohorts

The propensity score analysis indicates a statistically significant difference concerning the severity of adverse events between the treatment groups cefazolin and flucloxacillin (*p* = 0.019, stratified log-rank test applied to the sum of CTCAE ratings per patient). To illustrate these findings, boxplots were created, visualizing the difference in the severity of side effects between the two treatment groups across the PS strata (Fig. 1). The distinct differences observed in the distributions of side effects between cefazolin and flucloxacillin support the credibility and importance of the obtained results.

In seven patients, antibiotic treatment with flucloxacillin was discontinued due to side effects (13% of all patients receiving flucloxacillin). Treatment was changed to vancomycin in one patient due to leukopenia, and to cefazolin in six patients due to phlebitis (*n* = 4), elevated liver enzymes (*n* = 1) and nausea and vomiting (*n* = 1). Among the six patients whose treatment was changed from flucloxacillin to cefazolin due to AE, symptoms discontinued after the change in all but one patient with persistent nausea and vomiting. In no case cefazolin was discontinued due to AE (in one patient cefazolin was changed to flucloxacillin after one day of treatment without documented AEs at the time).

Overall, seven patients died before the 90-day-follow-up (*n* = 7/70, 10%), five of them while still hospitalized. One of the deaths was recorded in a patient receiving cefazolin only (*n* = 1/14, 7%) and six among patients receiving flucloxacillin only (*n* = 6/49, 12%). Recurring infection was noted in one of 14 patients (7%) with cefazolin and in two out of 38 (5%) with flucloxacillin.

## Discussion

In 13% of all patients receiving Flucloxacillin for treatment of MSSA BSI (*n* = 7) it was discontinued due to AE, which did not occur in patients receiving Cefazolin. A propensity score analysis indicated that AE were not only more frequent while receiving flucloxacillin than cefazolin, but that there was also a difference in severity of AE.

Consistent with previous data suggesting that cefazolin is at least as effective as ASP while severe AE are not more frequent [8–11], we observed a lower rate of AE with cefazolin as compared to flucloxacillin despite the small sample size, which may indicate a large difference in clinical tolerability. A propensity score analysis, conducted to reduce potential (selection) bias resulting from possible differences in patient characteristics between groups, indicates a statistically significant difference concerning the severity of AE. AE led to discontinuation of flucloxacillin in 13% of patients who received flucloxacillin (*n* = 7), confirming the high rates that have been reported previously [9, 11, 12]. Phlebitis and nausea each affected one third of patients receiving flucloxacillin and were the most common reasons to change treatment. Apart from leukopenia in one case, these AE were not life threatening, but still did cause such discomfort, that the clinical decision was taken to discontinue flucloxacillin.

No difference in treatment success at end of treatment and in recurring infection and mortality at 90 days was shown. However, case numbers and available covariates are too small to compare the efficacy of the two treatment strategies and do not allow to draw robust conclusions regarding outcomes, which is one of the main limitations of this study. While several reviews have supported increasing evidence that cefazolin is tolerated better than ASP while being at least as effective, they were limited by the retrospective nature and low quality of evidence of included studies (except for one prospective observational study from Korea [13]) [8, 9, 14]. The prospective design with weekly registration of AEs and 90-day follow-up data is a major strength of this study. Another strength is the use of advanced statistical methods (propensity score analysis).

## Conclusion

In line with several studies and meta-analyses published during recent years, this prospective observational study supports the use of cefazolin as a first line agent in MSSA BSI.

## References

[1] Gudiol F, Aguado JM, Almirante B, Bouza E, Cercenado E, Domínguez M (2015). Executive summary of the diagnosis and treatment of bacteremia and endocarditis due to *Staphylococcus aureus*. A clinical guideline from the Spanish Society of Clinical Microbiology and Infectious Diseases (SEIMC). Enferm Infecc Microbiol Clin.

[2] Baddour LM, Wilson WR, Bayer AS, Fowler VG, Tleyjeh IM, Rybak MJ (2015). Infective endocarditis in adults: diagnosis, antimicrobial therapy, and management of complications: a scientific statement for healthcare professionals from the American Heart Association. Circulation.

[3] Mermel LA, Allon M, Bouza E, Craven DE, Flynn P, O'Grady NP (2009). Clinical practice guidelines for the diagnosis and management of intravascular catheter-related infection: 2009 update by the Infectious Diseases Society of America. Clin Infect Dis.

[4] Lenhard JR, Bulman ZP (2019). Inoculum effect of β-lactam antibiotics. J Antimicrob Chemother.

[5] Bourreau A, Le Mabecque V, Broquet A, Caillon J (2023). Prevalence of a cefazolin inoculum effect associated with blaZ gene types, and clinical outcomes among methicillin-susceptible *Staphylococcus aureus* blood isolates of patients with infective endocarditis. Infect Dis Now.

[6] Antosz K, Battle S, Chang J, Scheetz MH, Al-Hasan M, Bookstaver PB (2023). Cefazolin in the treatment of central nervous system infections: a narrative review and recommendation. Pharmacotherapy.

[7] Pliakos EE, Ziakas PD, Mylonakis E (2021). The cost-effectiveness of cefazolin compared with Antistaphylococcal Penicillins for the treatment of methicillin-sensitive *Staphylococcus aureus* bacteremia. Open Forum Infect Dis.

[8] Weis S, Kesselmeier M, Davis JS, Morris AM, Lee S, Scherag A (2019). Cefazolin versus anti-staphylococcal penicillins for the treatment of patients with *Staphylococcus aureus* bacteraemia. Clin Microbiol Infect.

[9] Rindone JP, Mellen CK (2018). Meta-analysis of trials comparing cefazolin to antistaphylococcal penicillins in the treatment of methicillin-sensitive *Staphylococcus aureus* bacteraemia. Br J Clin Pharmacol.

[10] Tabah A, Laupland KB (2022). Update on *Staphylococcus aureus* bacteraemia. Curr Opin Crit Care.

[11] Shi C, Xiao Y, Zhang Q, Li Q, Wang F, Wu J (2018). Efficacy and safety of cefazolin versus antistaphylococcal penicillins for the treatment of methicillin-susceptible *Staphylococcus aureus* bacteremia: a systematic review and meta-analysis. BMC Infect Dis.

[12] Lecomte R, Bourreau A, Deschanvres C, Issa N, Le Turnier P, Gaborit B (2021). Comparative outcomes of cefazolin versus antistaphylococcal penicillins in methicillin-susceptible *Staphylococcus aureus* infective endocarditis: a post hoc analysis of a prospective multicentre French cohort study. Clin Microbiol Infect.

[13] Lee S, Song KH, Jung SI, Park WB, Lee SH, Kim YS (2018). Comparative outcomes of cefazolin versus nafcillin for methicillin-susceptible *Staphylococcus aureus* bacteraemia: a prospective multicentre cohort study in Korea. Clin Microbiol Infect.

[14] Bidell MR, Patel N, O'Donnell JN (2018). Optimal treatment of MSSA bacteraemias: a meta-analysis of cefazolin versus antistaphylococcal penicillins. J Antimicrob Chemother.

